# Correction: Linalool-based silver nanoconjugates as potential therapeutics for glioblastoma: *in silico* and *in vitro* insights

**DOI:** 10.1371/journal.pone.0336814

**Published:** 2025-11-17

**Authors:** Hina Manzoor, Muhammad Umer Khan, Samiullah Khan, Mohibullah Shah, Chaudhry Ahmed Shabbir, Hamad M. Alkhtani

Following the publication of this article [1], concerns were raised about the following:

- Details about the preparation and characterization of the nanoparticles are not included in the article.
- There are repeating areas within the graphs of Figs 12 and 13.

To address these concerns, the authors provide the following corrections.

The authors refer readers to their recent *PLOS One* paper [2], in which they have provided the full methodology of the production and characterization of the linalool-based silver nanoparticles used in both studies.

In reevaluating the data underlying Figs 12 and 13, the authors determined that the repetition observed in the RMSD and Rg plots was due to a typographical error in the CPPTRAJ input script, which inadvertently duplicated certain trajectory segments during analysis. To address this, the CPPTRAJ script was corrected and re-run on the original trajectories, producing new figures (corrected Figs 12 and 13, below) that now show continuous, non-repetitive profiles. These are consistent with the original data and do not materially differ in interpretation. The error stemmed solely from the input script and not from the molecular dynamics trajectories themselves.

The molecular dynamics simulations remain scientifically valid, and the corrected figures do not alter the overall analysis or conclusions of the study. Please note that Fig 13A and 13B have not been altered by the reanalysis.

The corrected CPPTRAJ input script and associated molecular dynamics dataset can be found at https://figshare.com/articles/dataset/b_MD_Data_b/30293731.

**Fig 12:**
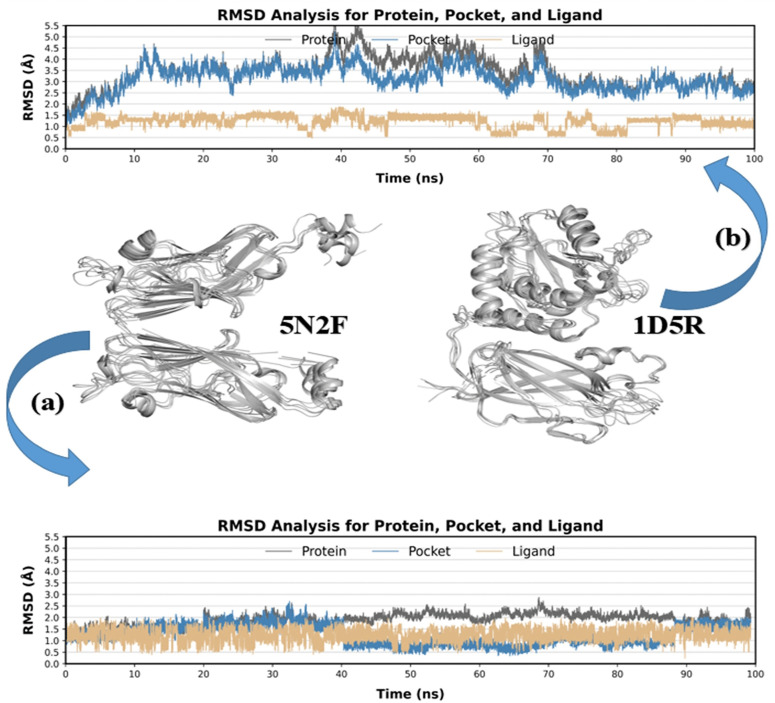
RMSD plots for linalool in complex with PD-L1 (a) and PTEN. **(b); from each docked complex’s individual 100 ns MD simulation trajectory, the ligand RMSD values were calculated as the protein-fit ligand.** Protein RMSD values were retrieved for the alpha carbon atoms.

**Fig 13:**
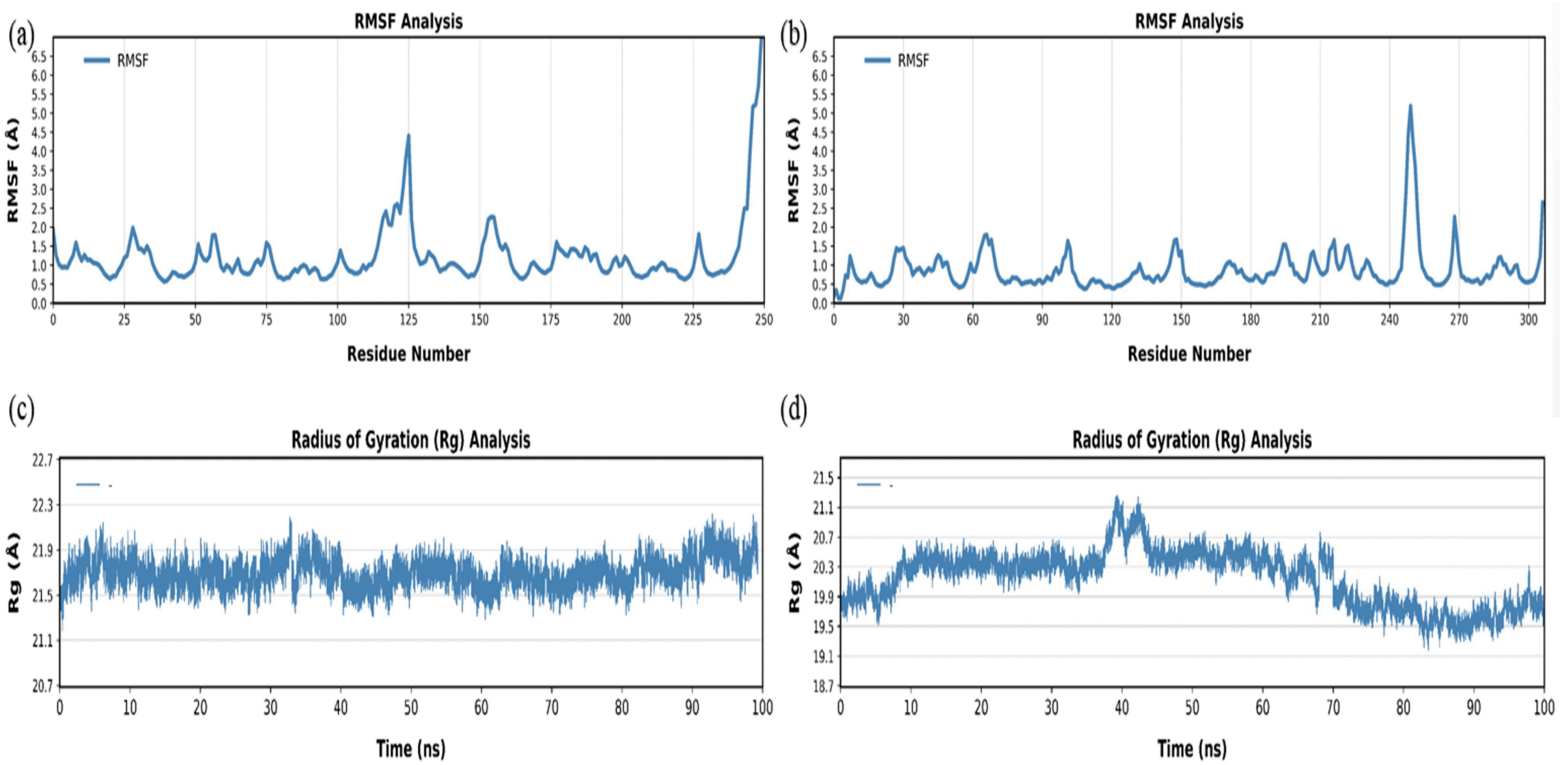
Root Mean Square Fluctuation (RMSF) and Radius of Gyration (Rg) analyses of protein-ligand complexes over a 100 ns molecular dynamics (MD) simulation. **(a, b)** RMSF plots showing the flexibility of each residue in the protein structures. **(c, d)** Rg plots showing compactness and structural stability over time. Panels (a) and (c) represent the PD-L1–linalool complex, whereas panels (b) and (d) correspond to the PTEN–linalool complex.
