# Supplementary material for: Linalool-based silver nanoconjugates as potential therapeutics for glioblastoma: in silico and in vitro insights
Source: PLoS One. 2025 Jun 12;20(6):e0325281. doi: 10.1371/journal.pone.0325281 (PMC12161535; doi:10.1371/journal.pone.0325281)
Supplement: S1 File — (DOCX) [file pone.0325281.s001.docx]

**Table S1.** List of 50 genes targets; immune checkpoint-related genes in group 1, and tumor suppressors/regulators in group 2.

| **Group 1** | **Group 2** |
| --- | --- |
| CD274 | PTEN |
| PDCD1 | TP53 |
| CTLA4 | RB1 |
| CDK4/6 6oql | NF1 |
| EGFR | MGMT |
| STAT3 | CDKN2A |
| VEGFA | BAX |
| HIF1A | FAS |
| FOXP3 | CASP8 |
| CXCL12 | GADD45A |
| CD276 | STK11 |
| MMP9 | SMAD4 |
| IL6 | PDCD4 |
| IL10 | DUSP6 |
| TGFBR1 | PRDM1 |
| NFKB1 | BCL2L11 |
| ZEB1 | NLRP3 |
| AKT1 | FADD |
| PIK3CA | TIGAR |
| FOXO1 | CD4 |
| ICAM1 | CD8A |
| ITGB3 | APC |
| TRAF2 | BID |
| PRKCB | MHC-II |
| BIRC5 | IRF1 |

**Table S2.** Human genes for head and neck cancer, a functional enrichment property of the STRING network

| **Gene** | **Z-score** |
| --- | --- |
| EGFR | 7.0 |
| TP53 | 6.9 |
| CD274 | 6.8 |
| CDKN2A | 6.6 |
| AKT1 | 6.4 |
| CD8A | 6.3 |
| CTLA4 | 6.2 |
| ERBB2 | 6.2 |
| PDCD1 | 6.1 |
| VEGFA | 6.1 |

**Table S3.** Human genes for U-87MG cell, a functional enrichment property of the STRING network

| **Gene** | **Z-score** |
| --- | --- |
| MGMT | 5.8 |
| EGFR | 5.7 |
| TP53 | 5.7 |
| AKT1 | 5.7 |
| CASP3 | 5.6 |
| ANXA5 | 5.5 |
| PTEN | 5.4 |
| EGF | 5.4 |
| PROM1 | 5.4 |
| PIK3CA | 5.3 |


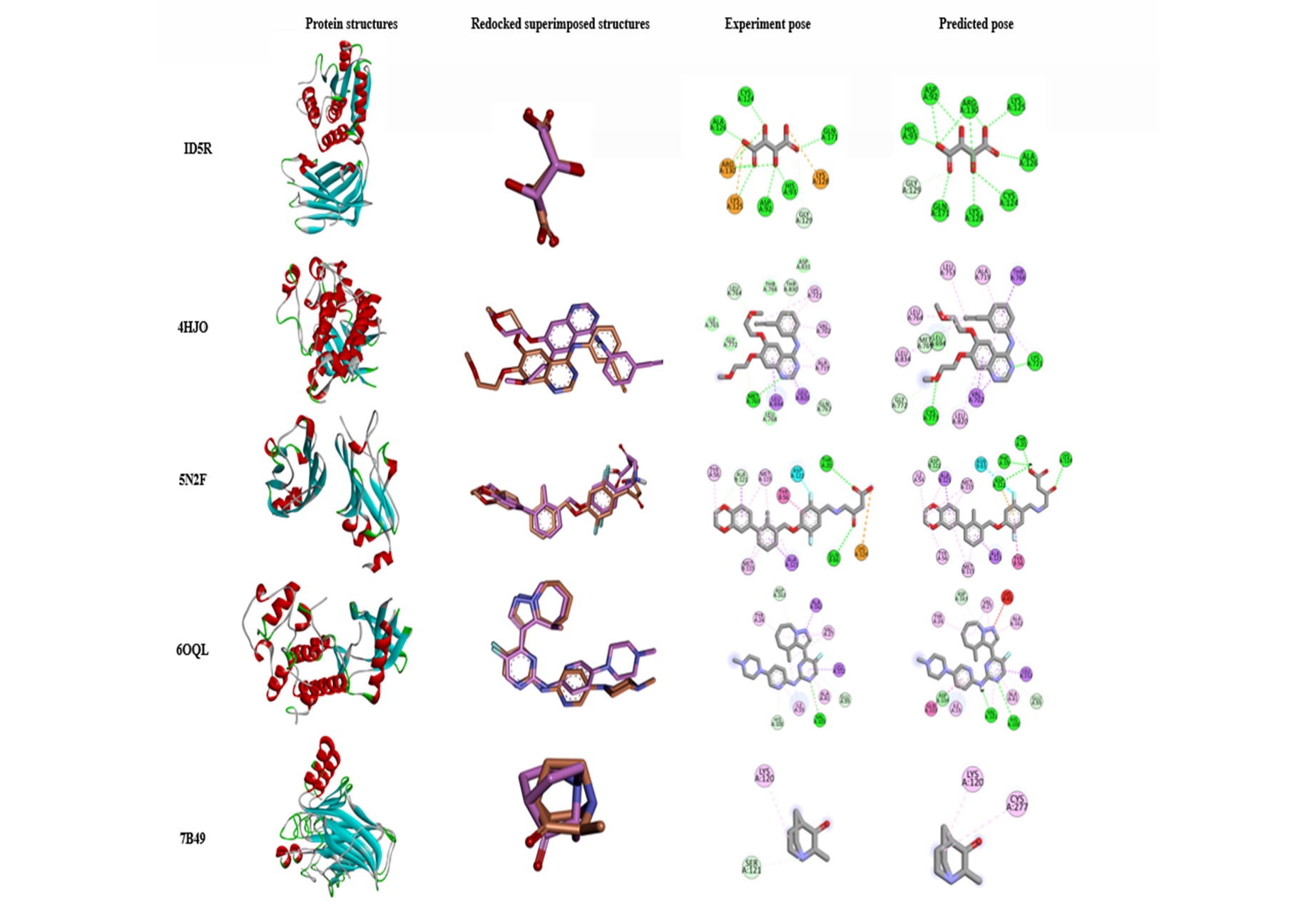


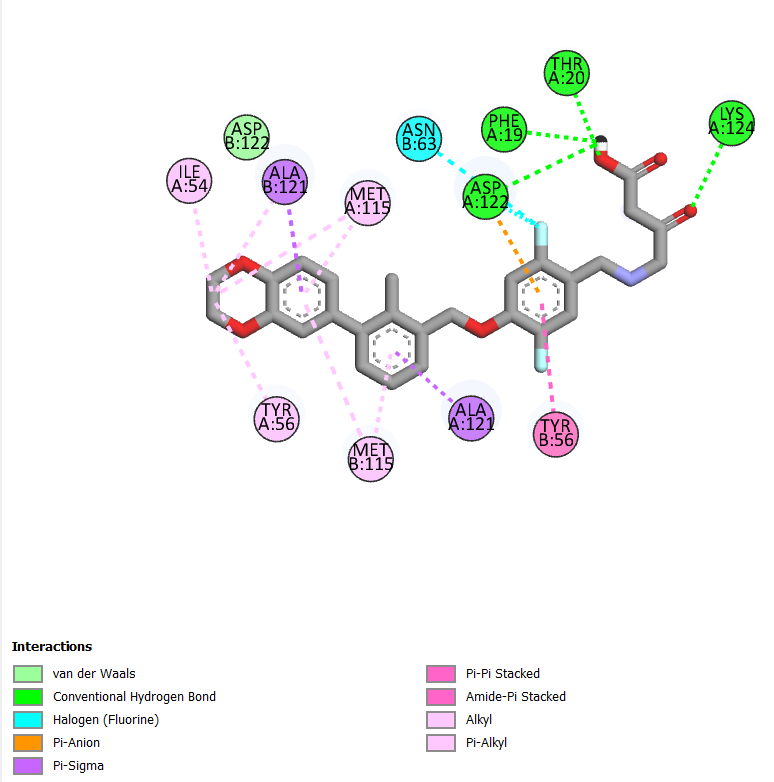


**Fig S1.** 3D structures of the target proteins along with redock-superimposed structures, crystallographic or experimental pose (orange), and their predicted structure pose (pink) and their 2D diagram showing interacting residues


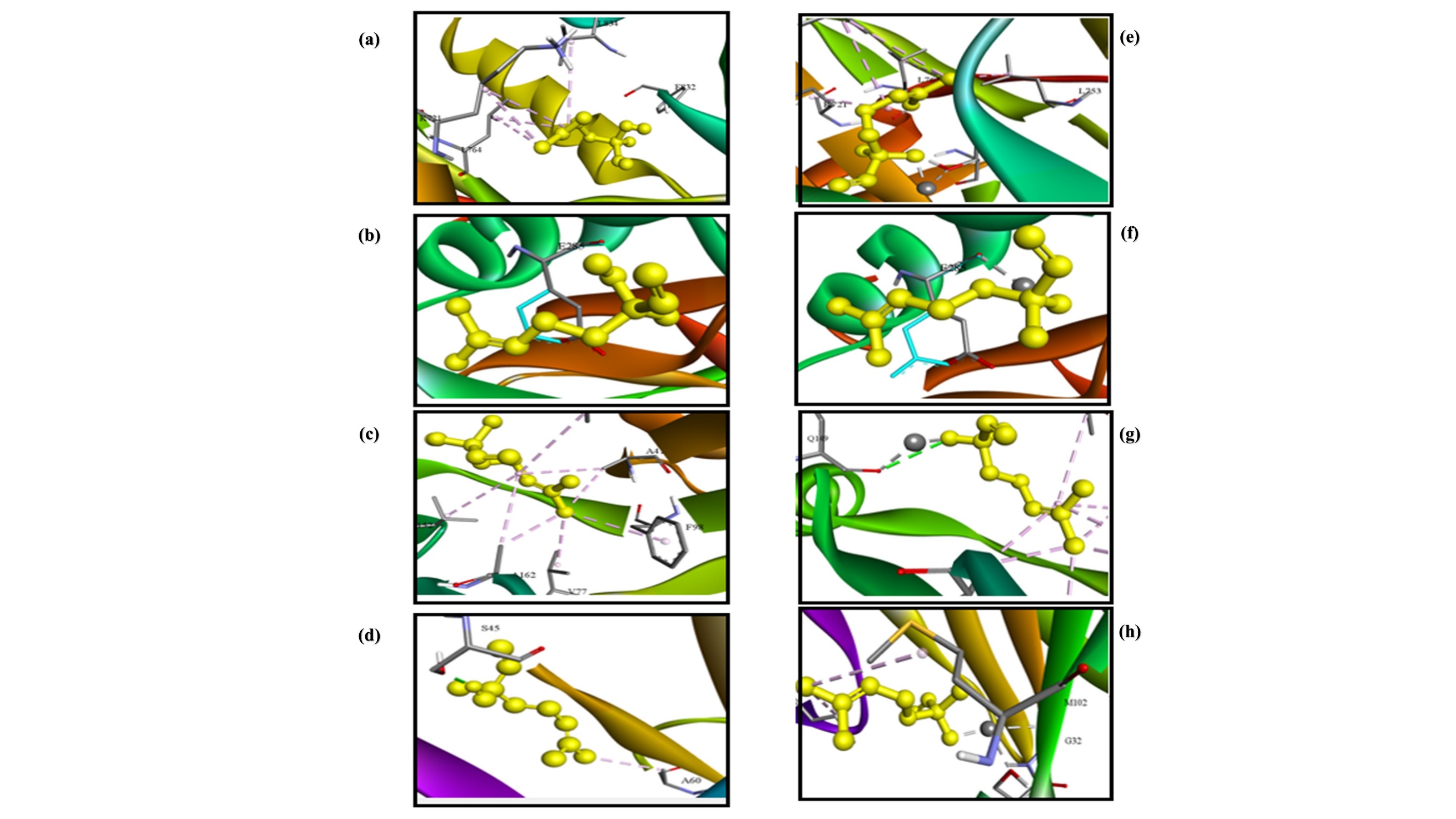


**Fig S2.** 3D representation of the binding mode of linalool with 4HJO, 7B49, 6OQL, 2HP4 (**a-d**) and LN@AgNPs with 4HJO, 7B49, 6OQL, 2HP4 (**e-h**)
